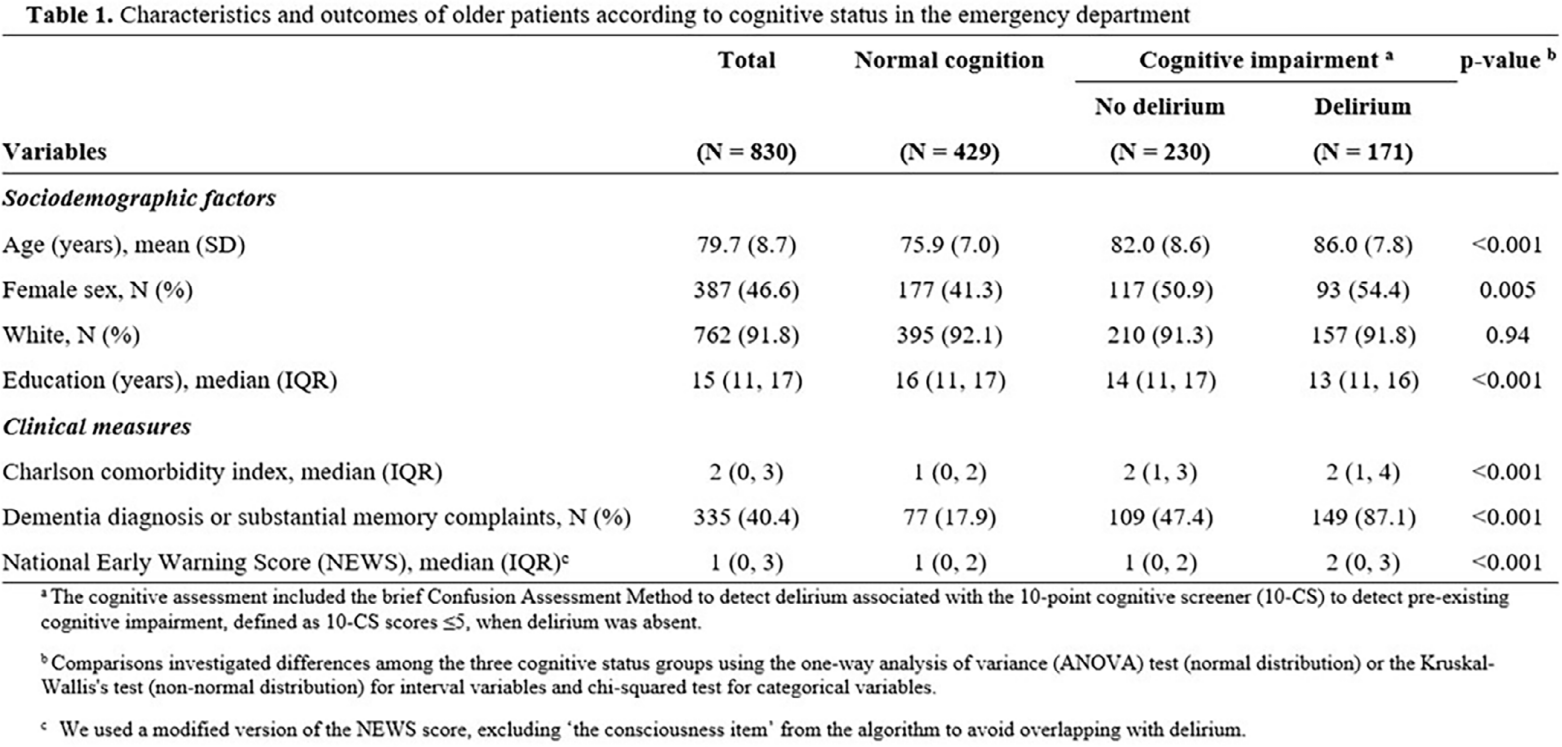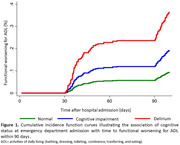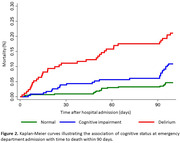# Fast and insightful: triage‐level cognitive screening captures risks beyond delirium in emergency care for older adults

**DOI:** 10.1002/alz.089508

**Published:** 2025-01-09

**Authors:** Gabriel Stanziola de Moraes, Pedro Kallas Curiati, Thiago J Avelino‐Silva, Christian Valle Morinaga, Claudia Kimie Suemoto, Márlon Juliano Romero Aliberti

**Affiliations:** ^1^ Hospital Sírio‐Libanês, São Paulo, São Paulo Brazil; ^2^ Marília Medical School, Marília, São Paulo Brazil; ^3^ University of California San Francisco, San Francisco, CA USA; ^4^ University of São Paulo Medical School, São Paulo, São Paulo Brazil

## Abstract

**Background:**

Although delirium is a powerful tool for identifying high‐risk older patients at the emergency department (ED), the feasibility and importance of cognitive screening beyond delirium remain debated in fast‐paced healthcare settings. We estimated the effect of comprehensive but pragmatic cognitive screening, capturing delirium and preexisting cognitive impairment, on predicting adverse outcomes within 90 days of admission in older adults at the ED.

**Method:**

We conducted a prospective cohort study comprising patients aged ≥65 years who were consecutively admitted to the ED of a large general hospital in Sao Paulo, Brazil. Trained healthcare professionals administered the brief Confusion Assessment Method (bCAM) and, for those without delirium, also the 10‐point Cognitive Screener (10‐CS), a practical 2‐minute tool validated for detecting preexisting cognitive impairment. Cognitive status was defined as normal (negative bCAM and 10‐CS>5), preexisting cognitive impairment (negative bCAM, but 10‐CS≤5), or delirium (positive bCAM). Investigators blinded to baseline data conducted telephone interviews to assess health outcomes, including functional decline in activities of daily living (ADL) and mortality over 90 days post‐admission. We used proportional hazards models to investigate associations between cognitive status and outcomes after adjusting for sociodemographic and clinical factors.

**Result:**

Among 830 patients (mean age = 80±9 years; women = 47%), 434 (52%) were classified as normal cognition and 396 (48%) as cognitive impairment (165 [20%] had delirium and 231 [28%] had preexisting cognitive impairment without delirium) (Table 1). Interestingly, 52% of patients with preexisting cognitive impairment without delirium had neither been diagnosed with dementia nor reported memory complaints. Patients with preexisting cognitive impairment without delirium, similar to those with delirium, showed an increased risk of functional decline in ADL (sub‐HR = 1.59; 95% CI = 1.01‐2.49) and mortality (HR = 2.19; 95% CI = 1.18‐4.09) within 90 days of admission, compared to patients with normal cognition (Figures 1‐2).

**Conclusion:**

In older patients at the ED, preexisting cognitive impairment without delirium emerges as a crucial risk factor for adverse outcomes, underscoring the need for comprehensive yet expedient cognitive screening in fast‐paced healthcare settings. The 10‐CS battery, swift and practical, is ideal for detecting frequently overlooked preexisting cognitive impairments, enabling the quick identification of high‐risk acutely ill older patients.